# Sonographic and Clinical Features of Papillary Thyroid Microcarcinoma Less than or Equal to Five Millimeters: A Retrospective Study

**DOI:** 10.1371/journal.pone.0148567

**Published:** 2016-02-16

**Authors:** Xingjian Lai, Bo Zhang, Yuxin Jiang, Jianchu Li, Ruina Zhao, Xiao Yang, Xiaoyan Zhang, Shenling Zhu, Qiong Wu, Sheng Cai, Yixiu Zhang

**Affiliations:** Department of Ultrasound, Chinese Academy of Medical Sciences & Peking Union Medical College Hospital, Beijing, China; Universite Libre de Bruxelles (ULB), BELGIUM

## Abstract

**Objective:**

To retrospectively compare the sonographic and clinical features of papillary thyroid microcarcinoma (PTMC) ≤5 mm and PTMC >5 mm to improve the diagnostic value of ultrasonography.

**Methods:**

A total of 367 cases of PTMC between January 2013 and December 2014 was included in this study. The patients were classified into group A (≤5 mm, n = 181) or group B (>5 mm, n = 186), and the sonographic and clinical features were reviewed and compared between the two groups.

**Results:**

There was no significant difference in the shape, ratio of length/width, boundary, peripheral halo ring, echogenicity, cystic change and accompanying Hashimoto's thyroiditis between these two groups. However, the calcification (61.3% vs. 72.6%) and hypervascularity (13.8% vs. 24.7%) were more frequent in group B (p = 0.026 and 0.008, respectively). The patients were younger, and more patients were aged less than 45 years (41.4% vs. 57.0%) in group B. Capsular invasion (7.2% vs. 34.4%), multifocality (21.5% vs. 48.9%), bilaterality (17.1% vs. 39.8%), central lymph node metastasis (13.8% vs. 38.2%) and lateral lymph node metastasis (1.1% vs. 5.4%) were more frequent in group B. No clinical or sonographic feature was related to cervical lymph node metastasis in group A, while less than 45 years in age (p = 0.010), male gender (p = 0.040), capsular invasion (p<0.001), multifocality (p = 0.016) and calcification (p = 0.042) were related to cervical lymph node metastasis in group B.

**Conclusions:**

The sonographic features of PTMC ≤5 mm were similar to those of PTMC >5 mm, including an irregular shape, a length/width ratio of ≥1, an unclear boundary, no peripheral halo ring, hypoechogenicity, no cystic change, calcification, no hypervascularity and no accompanying Hashimoto's thyroiditis. The clinical features of PTMC ≤5 mm were less aggressive than those of PTMC >5 mm.

## Introduction

Thyroid microcarcinoma is defined as thyroid carcinoma measuring ≤10 mm in its greatest dimension [1]. Papillary thyroid microcarcinoma (PTMC) is the most common form of thyroid microcarcinoma and is prevalent in the general population [2]. Several studies have reported that PTMC is detected in up to 35.6% of autopsy specimens, and up to 37.3% of PTMCs are associated with cervical lymph node metastasis; therefore, they exhibit aggressive behavior [2–4].

With the development of diagnostic technology, such as high-resolution ultrasonography and fine-needle aspiration biopsy, the diagnosis of PTMC measuring ≤5 mm in its greatest dimension has increased [5]. In the evaluation and surgical decision of PTMC ≤5 mm detected by ultrasonography with multifocality, although the clinical behavior of PTMC ≤5 mm is less aggressive than PTMC >5 mm, a higher risk of cervical lymph node metastasis should be considered [5]. Thus, it is important to diagnose PTMC ≤5 mm early by ultrasonography. However, to date, no literature has reported the sonographic features of PTMC ≤5 mm, and only a few studies have reported the clinical characteristics of PTMC ≤5 mm [5]. Therefore, the aim of the present study was to retrospectively compare the sonographic and clinical features of PTMC ≤5 mm and PTMC >5 mm to improve the diagnostic value of ultrasonography.

## Patients and Methods

### Patients

Peking Union Medical College Hospital ethics committee approved this retrospective study and written informed consent from the participants was waived. All of the records data and sonograms were de-identified and analyzed anonymously (S1 and S2 Files). The records of 375 consecutive patients who underwent surgery for primary PTMC confirmed by pathological examination at Peking Union Medical College Hospital between January 2013 and December 2014 were retrospectively reviewed. In eight cases, no nodule was detected by preoperative ultrasonography, and these patients were excluded from the analysis.

A total of 367 cases of PTMC with or without nodal metastasis was included in this study. Anterior compartment neck dissection was performed in all of the patients. If lateral nodal metastasis were suspected by ultrasonography or confirmed by fine needle aspiration biopsy, selective lateral level II-V neck dissection was conducted.

### Imaging and image analysis

Thyroid ultrasonography was performed using Philips iU22 (Philips Medical Systems, Bothell, WA, USA) or GE logic 9 (GE Healthcare, Wauwatosa, WI, USA) with a 5- to 12-MHz linear array transducer. Sonograms of the thyroid and the cervical lymph nodes were obtained in the transverse, longitudinal and oblique planes. All of the preoperative sonograms were interpreted by two experienced radiologists (B.Z. and XJ.L.) by consensus. Each of the radiologists had more than 9 years of thyroid sonography experience and was blinded to the clinical information.

For thyroid nodules, the number, the size, the shape, the ratio of length/width, the boundary, the peripheral halo ring, capsular invasion, echogenicity, calcification, cystic change, vascularity and accompanying disease were recorded. When a patient had multiple nodules, the largest one was used for sonographic feature analysis. The shape was determined as regular or irregular. The ratio of length/width was determined as <1 or ≥1. The boundary was determined as clear or unclear. The peripheral halo ring was determined as with or without peripheral halo ring. Capsular invasion was determined as with or without capsular invasion. Echogenicity was determined as hypoechogenicity, isoechogenicity or hyperechogenicity. Calcification was determined as no calcification, microcalcification or macrocalcification. Cystic change was determined as with or without cystic change. Vascularity was determined as hypervascularity (more than adjacent tissue), normal vascularity (similar to adjacent tissue) or avascularity (no blood flow) [6]. Accompanying disease was determined as with or without Hashimoto's thyroiditis.

### Statistical analysis

According to the tumor size, these patients were classified into “group A” consisting of 181 patients with a tumor size ≤5 mm and “group B” consisting of 186 patients with a tumor size ≤10 mm but >5 mm. The largest nodule was used for classification in a patient with multiple nodules [5].

Clinicopathologic factors, including gender, age, size of the tumor, tumor capsular invasion, multifocality, bilaterality and lymph node metastasis, and sonographic features, including shape, ratio of length/width, boundary, peripheral halo ring, echogenicity, calcification, cystic change, vascularity and accompanying disease, were compared between the two groups.

Statistical analyses were performed using the SPSS 11.5 software package (SPSS, Chicago, IL). Continuous variables were summarized as the means±SD, and categorical variables were summarized as percentages. χ^2^ test or independent *t* test was used as appropriate, and statistical significance was determined as a *p* value less than 0.05.

## Results

The clinical features of these patients are summarized in Table 1. There was no significant difference in the male-female ratio between these two groups. However, the patients were younger, and more patients were aged less than 45 years (41.4% vs. 57.0%) in group B. Capsular invasion (7.2% vs. 34.4%), multifocality (21.5% vs. 48.9%), bilaterality (17.1% vs. 39.8%), central lymph node metastasis (13.8% vs. 38.2%) and lateral lymph node metastasis (1.1% vs. 5.4%) were more frequent in group B.

**Table 1:** Comparison of clinical features between the two groups.

Characteristic	Group A (n = 181)	Group B (n = 186)	*p* value
**Size of tumor (mm)**	3.9±0.1	7.4±0.1	<0.001
**Age, mean±SD**^a^ **(years)**	46.6±8.3	43.0±11.2	0.001
**Less than 45 years**			0.003
**Yes**	75 (41.4%)	106 (57.0%)	
**No**	106 (58.6%)	80 (43.0%)	
**Gender**			0.394
**Male**	40 (22.1%)	49 (26.3%)	
**Female**	141 (77.9%)	137 (73.7%)	
**Tumor capsular invasion**			<0.001
**No**	168 (92.8%)	122 (65.6%)	
**Yes**	13 (7.2%)	64 (34.4%)	
**Multifocality**			<0.001
**No**	142 (78.5%)	95 (51.1%)	
**Yes**	39 (21.5%)	91 (48.9%)	
**Bilaterality**			<0.001
**No**	150 (82.9%)	112 (60.2%)	
**Yes**	31(17.1%)	74 (39.8%)	
**Central lymph node metastasis**			<0.001
**No**	156 (86.2%)	115 (61.8%)	
**Yes**	25 (13.8%)	71 (38.2%)	
**Lateral lymph node metastasis**			0.036
**No**	179 (98.9%)	176 (94.6%)	
**Yes**	2 (1.1%)	10 (5.4%)	

^a^SD = standard deviation.

**Fig 1:**
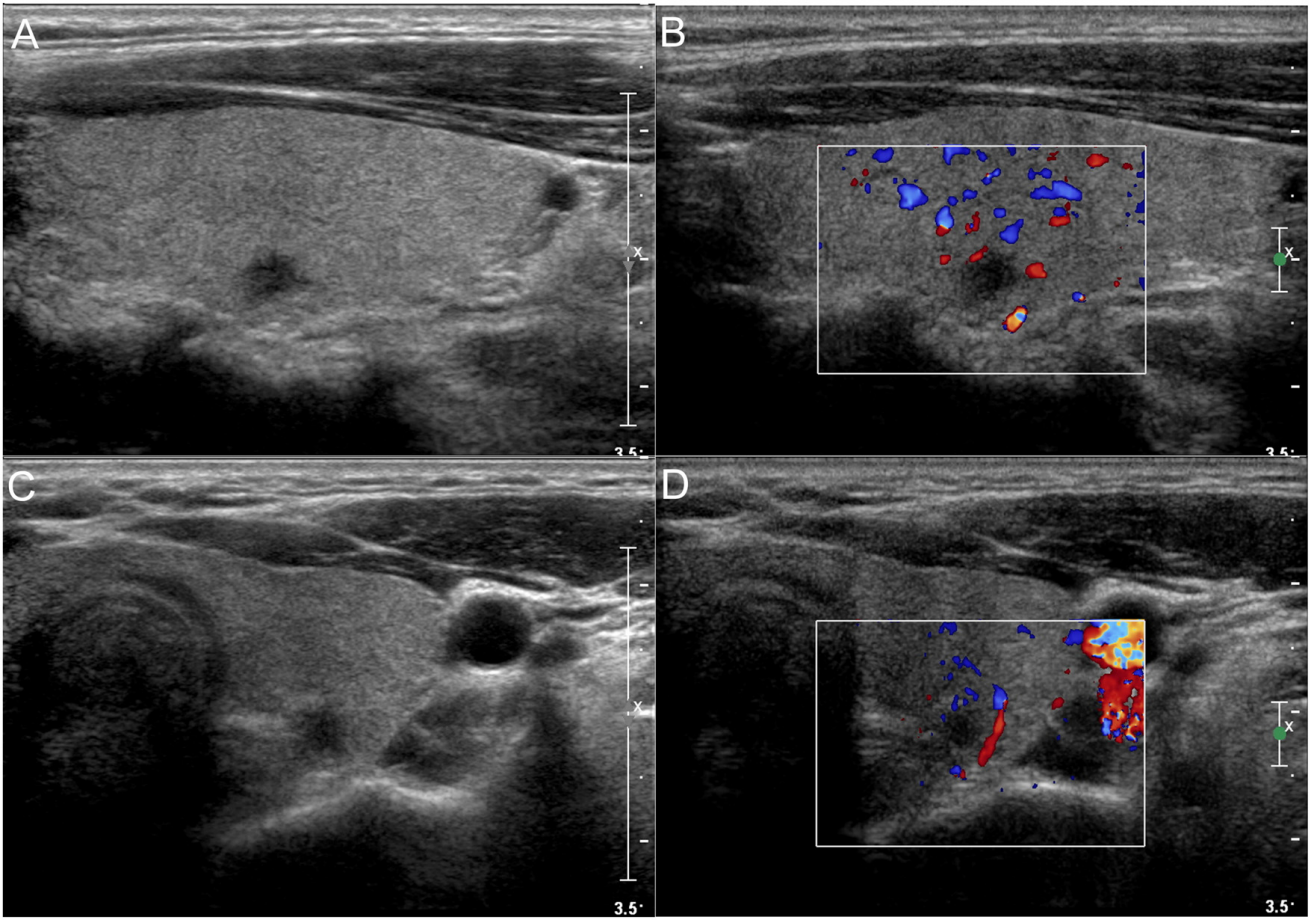
A 38-year-old female patient with papillary thyroid microcarcinoma less than or equal to 5 mm. (a) The longitudinal gray scale sonogram shows an irregular shape, a length/width ratio of ≥1, an unclear boundary, no peripheral halo ring, hypoechogenicity, no cystic change and no accompanying Hashimoto's thyroiditis. (b) The longitudinal color sonogram shows no hypervascularity. (c) The transverse gray scale sonogram shows an irregular shape, a length/width ratio of ≥1, an unclear boundary, no peripheral halo ring, hypoechogenicity, no cystic change and no accompanying Hashimoto's thyroiditis. (d) The transverse color sonogram shows no hypervascularity.

The sonographic features of these patients are summarized in Table 2. Most of the nodules in group A had an irregular shape (89.0%), a length/width ratio ≥1 (76.8%), an unclear boundary (91.2%), no peripheral halo ring (99.4%), hypoechogenicity (98.3%), no cystic change (93.9%), calcification (61.3%), no hypervascularity (86.2%) and no accompanying Hashimoto's thyroiditis (74.0%) (Fig 1). There was no significant difference in the shape, the ratio of length/width, the boundary, the peripheral halo ring, echogenicity, cystic change and accompanying Hashimoto's thyroiditis between these two groups. However, the calcification (61.3% vs. 72.6%) and hypervascularity (13.8% vs. 24.7%) were more frequent in group B (p = 0.026 and 0.008, respectively).

**Table 2:** Comparison of sonographic features between the two groups.

Characteristic	Group A (n = 181)	Group B (n = 186)	*p* value
**Shape**			0.733
**Regular**	20 (11.0%)	18 (9.7%)	
**Irregular**	161 (89.0%)	168 (90.3%)	
**Ratio of length/width**			0.157
**<1**	42 (23.2%)	56 (30.1%)	
**⩾1**	139 (76.8%)	130 (69.9%)	
**Boundary**			0.311
**Clear**	16 (8.8%)	23 (12.4%)	
**Unclear**	165 (91.2%)	163 (87.6%)	
**Peripheral halo ring**			0.122
**No**	180 (99.4%)	180 (96.8%)	
**Yes**	1 (0.6%)	6 (3.2%)	
**Echogenicity**			0.140
**Hypoechogenicity**	178 (98.3%)	177 (95.2%)	
**Isoechogenicity**	3 (1.7%)	9 (4.8%)	
**Hyperechogenicity**	0	0	
**Cystic change**			0.651
**No**	170 (93.9%)	177 (95.2%)	
**Yes**	11 (6.1%)	9 (4.8%)	
**Calcification**			0.072
**No calcification**	70 (38.7%)	51 (27.4%)	
**Microcalcification**	103 (56.9%)	125 (67.2%)	
**Macrocalcification**	8 (4.4%)	10 (5.4%)	
**Presence of calcification**			0.026
**No**	70 (38.7%)	51 (27.4%)	
**Yes**	111 (61.3%)	135 (72.6%)	
**Vascularity**			0.022
**Avascularity**	36 (19.9%)	27 (14.5%)	
**Normal vascularity**	120 (66.3%)	113 (60.8%)	
**Hypervascularity**	25 (13.8%)	46 (24.7%)	
**Presence of hypervascularity**			0.008
**No**	156 (86.2%)	140 (75.3%)	
**Yes**	25 (13.8%)	46 (24.7%)	
**Accompanying HT**^a^			0.905
**No**	134 (74.0%)	139 (74.7%)	
**Yes**	47 (26.0%)	47 (25.3%)	

^a^HT = Hashimoto's thyroiditis.

No clinical or sonographic feature was related to cervical lymph node metastasis in group A (Tables 3 and 4), while less than 45 years in age (p = 0.010), male (p = 0.040), capsular invasion (p<0.001), multifocality (p = 0.016) and calcification (p = 0.042) were related to cervical lymph node metastasis in group B (Tables 5 and 6).

**Table 3:** Clinical features related to lymph node metastasis in group A.

Characteristic	No. of patients (n = 181)^a^	LN metastasis (n = 25)^b^	*p* value
**Less than 45 years**			0.279
**Yes**	75	13 (17.3%)	
**No**	106	12 (11.3%)	
**Gender**			0.443
**Male**	40	7 (17.5%)	
**Female**	141	18 (12.8%)	
**Tumor capsular invasion**			0.085
**No**	168	21 (12.5%)	
**Yes**	13	4 (30.8%)	
**Multifocality**			1.000
**No**	142	20 (14.1%)	
**Yes**	39	5 (12.8%)	
**Bilaterality**			0.775
**No**	150	20 (13.3%)	
**Yes**	31	5 (16.1%)	

^a^No. = number.

^b^LN = lymph node.

**Table 4:** Sonographic features related to lymph node metastasis in group A.

Characteristic	No. of patients (n = 181)^a^	LN metastasis (n = 25)^b^	*p* value
**Shape**			1.000
**Regular**	20	2 (10.0%)	
**Irregular**	161	23 (14.3%)	
**Ratio of length/width**			0.072
**<1**	42	2 (4.8%)	
**⩾1**	139	23 (16.5%)	
**Boundary**			0.702
**Clear**	16	1 (6.3%)	
**Unclear**	165	24 (14.5%)	
**Peripheral halo ring**			1.000
**No**	180	25 (13.9%)	
**Yes**	1	0	
**Echogenicity**			1.000
**Hypoechogenicity**	178	25 (14.0%)	
**Isoechogenicity**	3	0	
**Cystic change**			0.651
**No**	170	23 (13.5%)	
**Yes**	11	2 (18.2%)	
**Presence of calcification**			0.275
**No**	70	7 (10.0%)	
**Yes**	111	18 (16.2%)	
**Presence of hypervascularity**			1.000
**No**	156	22 (14.1%)	
**Yes**	25	3 (12.0%)	
**Accompanying HT**^c^			1.000
**No**	134	19 (14.2%)	
**Yes**	47	6 (12.8%)	

^a^No. = number.

^b^LN = lymph node.

^c^HT = Hashimoto's thyroiditis.

**Table 5:** Clinical features related to lymph node metastasis in group B.

Characteristic	No. of patients (n = 186)^a^	LN metastasis (n = 71)^b^	*p* value
**Less than 45 years**			0.010
**Yes**	106	49 (46.2%)	
**No**	80	22 (27.5%)	
**Gender**			0.040
**Male**	49	25 (51.0%)	
**Female**	137	46 (33.6%)	
**Tumor capsular invasion**			<0.001
**No**	122	35 (28.7%)	
**Yes**	64	36 (56.3%)	
**Multifocality**			0.016
**No**	95	28 (29.5%)	
**Yes**	91	43 (47.3%)	
**Bilaterality**			0.090
**No**	112	37 (33.0%)	
**Yes**	74	34 (45.9%)	

^a^No. = number

^b^LN = lymph node.

**Table 6:** Sonographic features related to lymph node metastasis in group B.

Characteristic	No. of patients (n = 186)^a^	LN metastasis (n = 71)^b^	*p* value
**Shape**			0.800
**Regular**	18	6 (33.3%)	
**Irregular**	168	65 (38.7%)	
**Ratio of length/width**			0.870
**<1**	56	22 (39.3%)	
**⩾1**	130	49 (37.7%)	
**Boundary**			0.821
**Clear**	23	8 (34.8%)	
**Unclear**	163	63 (38.7%)	
**Peripheral halo ring**			1.000
**No**	180	69 (38.3%)	
**Yes**	6	2 (33.3%)	
**Echogenicity**			1.000
**Hypoechogenicity**	177	68 (38.4%)	
**Isoechogenicity**	9	3 (33.3%)	
**Cystic change**			1.000
**No**	177	68 (38.4%)	
**Yes**	9	3 (33.3%)	
**Presence of calcification**			0.042
**No**	51	13 (25.5%)	
**Yes**	135	58 (43.0%)	
**Presence of hypervascularity**			0.294
**No**	140	50 (35.7%)	
**Yes**	46	21 (45.7%)	
**Accompanying HT**^c^			0.118
**No**	139	58 (41.7%)	
**Yes**	47	13 (27.7%)	

^a^No. = number.

^b^LN = lymph node.

^c^HT = Hashimoto's thyroiditis.

## Discussion

Currently, thyroid nodules are primarily evaluated by ultrasonography and fine-needle aspiration biopsy. For suspected thyroid nodules, though cytology is an effective diagnostic method, invasiveness limited the clinical application [7]. Ultrasonography is a convenient, cost effective, highly sensitive and noninvasive preoperative diagnostic method, and it has been the preferred screening method [8].

Several studies have shown that certain sonographic features of a thyroid nodule and a combination of features have high predictive value for malignancy, including a shape taller than the width, irregular infiltrative margins, an absent halo, nodule hypoechogenicity, the presence of microcalcifications, increased intranodular vascularity and the presence of suspicious cervical lymphadenopathy, regardless of nodule size [9,10]. In our study, most cases of PTMC ≤5 mm had an irregular shape, a length/width ratio of ≥1, an unclear boundary, no peripheral halo ring, hypoechogenicity, no cystic change, calcification, no hypervascularity and no accompanying Hashimoto's thyroiditis, and the sonographic features of PTMC ≤5 mm were similar to those of PTMC >5 mm.

However, calcification (61.3% vs. 72.6%) and hypervascularity (13.8% vs. 24.7%) were more frequent in PTMC >5 mm, demonstrating that calcification and vascularity increased as the size increased. Similar to our results, Moon et al. reported that fewer malignant nodules ≤10 mm had microcalcification than larger nodules, and the diagnostic value of microcalcification was greater for large nodules than for small nodules [9]. In some nodules ≤10 mm, it might be difficult to differentiate microcalcifications from colloid crystals, and this may be an explanation for why calcification was more common in PTMC >5 mm [7].

In our study, central lateral lymph node metastasis (13.8% vs. 38.2%) and lateral lymph node metastasis (1.1% vs. 5.4%) were more frequent in PTMC >5 mm, revealing that the clinical features of PTMC ≤5 mm were less aggressive than those of PTMC >5 mm. In our study, capsular invasion (7.2% vs. 34.4%), multifocality (21.5% vs. 48.9%) and bilaterality (17.1% vs. 39.8%) were also more frequent in PTMC >5 mm, findings that were consistent with those previously reported [11–13]. Therefore, a size >5 mm is an aggressive risk factor of PTMC [14].

Some authors suggested that an incidentally detected PTMC <5 mm should not be classified as a carcinoma, it is an occult papillary tumor and treatment is not necessary [15]. Because the sizes of most PTMCs change little during long-term follow up, and the occurrence of metastasis is infrequent, some authors suggested that continuous observation only is necessary [16]. These results may coincide with the results of our study. In our series, no clinical factor or sonographic feature was related to cervical lymph node metastasis in PTMC ≤5 mm. However, some authors have shown that the recurrence rate of PTMC ≤5 mm and PTMC >5 mm had no significant difference [17,18]. In our study, central lateral lymph node metastasis occurred in 13.8% of PTMC ≤5 mm, and lateral lymph node metastasis occurred in 1.1% of PTMC ≤5 mm. Thus, in PTMC ≤5 mm, the significance of cervical lymph node metastasis should not be overlooked [5].

In our study, less than 45 years in age, male gender, capsular invasion, multifocality and calcification were identified as independent predictive factors of cervical lymph node metastasis in PTMC >5 mm, findings that were consistent with those previously reported. Zhao et al. reported that age, gender, local infiltration and multifocality were all independent correlates of lateral lymph node metastasis in PTMC [3]. Kwak et al. reported that there was a statistically significant association between lateral lymph node metastasis of PTMC and the presence of calcification on ultrasonography [19].

There are several limitations in our study. First, the current study is a retrospective study and real-time evaluation of ultrasonography findings is impossible. Thus, the interpretation may vary among different operators. However, all of the preoperative sonograms were interpreted by two experienced radiologists by consensus. Second, selective ipsilateral level II-V neck dissection for lateral nodal metastasis was conducted only in cases that were suspected by ultrasonography or confirmed by fine needle aspiration biopsy. As a result, lateral nodal metastasis may have been underestimated. However, this limitation cannot weaken the importance of this study because prophylactic dissection of nonpalpable lymph nodes is not beneficial in PTMC patients [20].

In conclusion, the sonographic features of PTMC ≤5 mm were similar to those of PTMC >5 mm, including an irregular shape, a length/width ratio of ≥1, an unclear boundary, no peripheral halo ring, hypoechogenicity, no cystic change, calcification, no hypervascularity and no accompanying Hashimoto's thyroiditis. However, the calcification and hypervascularity were more frequent in PTMC >5 mm. The clinical features of PTMC ≤5 mm were less aggressive than those of PTMC >5 mm, and capsular invasion, multifocality, bilaterality and lymph node metastasis were more frequent in PTMC >5 mm.

## Supporting Information

S1 File The data of all cases. (XLSX)

S2 File STROBE statement-checklist of items that should be included in reports of case-control studies. (DOC)
